# Fish Meal Replacement and Early Mild Stress Improve Stress Responsiveness and Survival of Fish after Acute Stress

**DOI:** 10.3390/ani13081314

**Published:** 2023-04-11

**Authors:** Mahyar Zare, Mohammad Kazempour, Hossein Hosseini, Seyedeh Mahsa Hosseini Choupani, Sobhan R. Akhavan, Artur Rombenso, Noah Esmaeili

**Affiliations:** 1 South Bohemian Research Center of Aquaculture and Biodiversity of Hydrocenoses, Faculty of Fisheries and Protection of Waters, Institute of Aquaculture and Protection of Waters, University of South Bohemia in České Budějovice, Husova třída, 37005 České Budějovice, Czech Republic; 2 Department of Microbiology, Pathobiology & Basic Sciences, Faculty of Veterinary Medicine, Razi University, Kermanshah 6714414971, Iran; 3 Department of Fisheries, Faculty of Marine Science, Tarbiat Modares University, Noor 4641776489, Iran; 4 Nelson Marlborough Institute of Technology, 322 Hardy Street, Private Bag 19, Nelson 7010, New Zealand; 5 Commonwealth Scientific and Industrial Research Organisation CSIRO, Agriculture and Food, Livestock & Aquaculture Program, Bribie Island Research Centre, Woorim, QLD 4507, Australia; 6 Institute for Marine and Antarctic Studies, University of Tasmania, Hobart, TAS 7053, Australia

**Keywords:** antioxidant responses, blood performance, blood biochemistry, stress physiology, stress responses

## Abstract

**Simple Summary:**

Early mild stress is a less-studied topic in aquaculture. Due to limitations in fish meal and oil resources as an obstacle for aquaculture sustainability, fish meal replacement has been widely investigated in different aquatic species but not oscars. This study investigated the effect of early mild stress (netting) and fishmeal replacement with meat and bone meal and their interactions on growth, hematology, blood biochemistry, immune responses, antioxidant system, liver enzymes, and stress responses of oscars. After the experiment, FM levels in diets did not affect growth data, but the survival rate after the acute confinement stress was lower in those fed not-enough fish meals. Further, exposing fish to too much early mild stress decreased growth and survival rate. Lower survival and growth rate in those treatments connected to the lowest blood performance, total protein, lysozyme, complement C4, complement C3, immuno-globulin, superoxide dismutase, catalase, glutathione peroxidase, and the highest glucose.

**Abstract:**

Stress responsiveness and fish meal (FM) replacement are two of the most important concerns toward achieving sustainable aquaculture. The purpose of this study was to see how early mild stress (netting) and FM replacement with meat and bone meal (MBM) affected oscar (*Astronotus ocellatus*; 5.2 ± 0.9 g) growth, hematology, blood biochemistry, immune responses, antioxidant system, liver enzymes, and stress responses. Oscars were subjected to a 3 × 3 experimental design (three fish meal replacement levels: 250, 180 and 110 g/kg of FM in diets; three stress periods: 0-, 2- and 3-times early mild stress). After ten weeks of the experiment, FM levels in diets did not affect growth data, but the survival rate after the acute confinement (AC) stress was lower in 11FM treatments (47.7% compared to 67.7%) than others. Fish exposed to the 3Stress schedule had a lower growth (31.03 ± 6.50 g) and survival rate (55.5%) after the AC stress than the 2Stress group (38.92 ± 6.82 g and 70.0%). Lower survival and growth rate in the 3Stress and 11FM groups coincided with the lowest blood performance, total protein, lysozyme, complement C4, complement C3, immunoglobulin, superoxide dismutase, catalase, glutathione peroxidase, and the highest glucose, cortisol, low-density lipoprotein and aspartate aminotransferase serum levels. Altogether, this study revealed that it is possible to replace FM with MBM up to 28% (180 g/kg of FM) without negative effects on the growth and health of juvenile oscar as dietary 110 g/kg of FM impaired fish health. While fish welfare should be considered, we can conclude that mild stress (2Stress) during the farming period, but without adding excessive alternative protein sources, can improve the stress responsiveness of oscar.

## 1. Introduction

Aquaculture is becoming a leader in food sectors due to providing relatively sustainable protein for humans. Although most of the aquaculture focus has been on edible aquatic species, the ornamental fish market has become one of the most profitable sectors in aquaculture [1]. However, many factors, including, but not limited to, the availability of marine proteins and oils, disease outbreaks and stress can affect the sustainability of the ornamental fish market [2].

Fish meal (FM) and fish oil are limited resources in the marine environment. Therefore, any action that can reduce their usage in diets without a negative impact on animals’ growth and health can be a step toward the sustainability of aquaculture and ornamental fish market. FM can directly affect growth as well as the immune system and stress response of carnivorous species. While insufficient FM levels in diets can impair growth and health [3,4,5,6], excessive FM in diets is not reasonable and less sustainable. Numerous studies in aquaculture nutrition are related to FM and fish oil replacement; a wide range of ingredients have been tested, such as plant, microbe, algae and animal-based protein alternatives [7,8,9,10,11,12]. Among the alternative proteins, animal-based proteins such as meat and bone meal (MBM), poultry by-product meal and blood meal (BM) have been successfully used in fish diets due to high protein content, favourable amino acid profile, high digestibility of nutrients, low levels of fibre, starch and non-soluble carbohydrates [7]. For example, FM was replaced with MBM up to 40% in Ussuri catfish (*Pseudobagrus ussuriensis*), and 45% in yellow croaker (*Pseudosciaena crocea*) [13] and rainbow trout (*Oncorhynchus mykiss*) [14]. However, higher levels negatively affect growth and immunity. MBM has a deficiency of lysine and methionine, and therefore, it is suggested to supplement these two amino acids to diets when FM is replaced with MBM [15,16]. BM is a rich source of lysine but cannot be included in diets at high levels (less than 100 g/kg diets can be used for different fish species) as its amino acids content is imbalanced.

Stress is a crucial factor that affects the profitability and long-term sustainability of any aquaculture system. This parameter is much more important in ornamental fish species such as oscar (*Astronotus ocellatus*), which is usually farmed in high density. Mild stress is defined as a situation whereby fish do not die or suffer significantly from stress, and fish have a short-term response. In the rare early mild stress (EMS) studies in aquatic species, lower hypothalamus catecholaminergic and brainstem serotonergic responses to stress and cortisol responsiveness were observed [17,18,19]. However, there is a paucity of information about applying regular scheduled early stress and investigating physiological changes after applying acute stress. In our previous research, the early stress response was tested in combination with different levels of lipids [2] and proteins [20]. Esmaeili et al. (2022) showed that fish exposed to just two weeks of EMS during ten weeks of the experiment had the highest survival rate after AC stress. Further, treatments that were fed 180 g/kg of lipid in their diets had a higher survival rate after AC stress than those fed 100 g/kg of lipid in their diets. Zare et al. (2022) indicated that oscar fed 510 g/kg of protein in the diet and exposed to two weeks of EMS had the highest survival rate compared to those fed 380 g/kg of protein and/or four weeks of EMS during ten weeks of the experiment. These results showed that two weeks of EMS resulted in the highest survival rate regardless of lipid or protein contents in diets. In previous research, two- and four-week stress were applied [2,20]. Therefore, as follow-up research, two and three weeks of stress for the present study were tested with three levels of FM inclusion (250, 180 and 110 g/kg of FM in diets).

Oscar is a highly traded ornamental fish species because of its early maturation, high fecundity, colourful skin, behaviour and interaction with their owners [2]. This species is carnivorous and requires a high level of FM and fish oil in its diet. However, based on the Scopus database, no study investigated FM replacement in this fish species. Further, no investigation has tested the interaction of EMS and FM replacement in aquatic species concurrently. Therefore, this study was conducted to examine the effect of FM replacement with MBM and their interactions with EMS on the growth performance, haematology, blood biochemistry, immune response, antioxidant activities, stress response and liver enzymes of juvenile oscar.

## 2. Materials and Methods

### 2.1. Animal Ethic, Animal and Experimental Conditions

The National Animal Care and Committee approved all experimental protocols (281-1385). Six hundred forty-eight fish were used in this study (initial weight: 5.2 ± 0.9 g) obtained from the Abzian Center (Mahallat, Markazi, Iran). Oscars were adapted to experimental conditions for 14 days using a commercial diet (500 and 150 g/kg crude protein and lipid, respectively). Twenty-seven rectangular glass tanks (100 L), three aquaria per treatment and twenty-four oscar per tank were used in this experiment. Fish were fed with the experimental diets three times a day (09:00, 14:00 and 19:00) for ten weeks to apparent satiation levels. The photoperiod was set to 12D:12L. During the experiment, to remove faeces and debris, 20–30% of each tank’s water was changed daily with dechlorinated water. Water quality parameters were checked on a regular basis and kept at standard levels through aeration with compressed air and filtration, which was detailed in our previous study [2,20].

### 2.2. Diet Formulation and Experimental Design

Three isonitrogenous (450 g of crude protein/kg feed) diets with the same lipid levels (180 g crude lipid/kg feed) (Table 1) were formulated by Lindo software. To find the optimum protein levels for oscar, 380, 420, 460 and 500 g/kg of protein were tested, and 450 g/kg was the optimum level (unpublished data). Therefore, the protein level was adjusted to 450 g/kg for the present study. FM in diets was replaced with MBM. Corn meal and BM were used to level the nitrogen equivalent among treatments with a maximum 3% change across diets. A nearby Animal & Aquatic Feed store (Arak, Markazi, Iran) supplied the diet ingredients.

Three scheduled stress windows (this involved dragging an aquarium net around the tank for 5 min after a water exchange without actively chasing or removing any fish) on Monday and Friday of the proposed weeks were tested during the ten-week experimental period. This schedule has already been tested in previous studies [2,20], and a new schedule (three weeks) was tested as four-week stress was too much and negatively affected fish physiology and survival after acute stress [20].

Therefore, the nine experimental treatments were 25FM0Stress (250 g/kg of FM in diet and without stress), 25FM2Stress (250 g/kg of FM in diet and stress in weeks two and eight), 25FM3Stress (250 g/kg of FM in diet and stress in weeks two, six and eight), 18FM0Stress (180 g/kg of FM in diet and without stress), 18FM2Stress (180 g/kg of FM in diet and stress in weeks two and eight), 18FM3Stress (250 g/kg of FM in diet and stress in weeks two, six and eight), 11FM0Stress (110 g/kg of FM in diet and without stress), 11FM2Stress (110 g/kg of FM in diet and stress in weeks six and eight) and 11FM3Stress (110 g/kg of FM in diet and stress in weeks two, six and eight) (Table 2). The dietary ingredients were dried, mixed, pelleted and stored based on our previous studies [2,21].

### 2.3. Collecting Samples and Measuring Growth Performance

Prior to final measurements (end of week 9), fish were starved for 24 h and anaesthetised using the clove oil stock solution (50 ppm) [22]. At the end of the experiment, weight gain, specific growth rate (SGR), feed conversion ratio (FCR), daily feed intake (DFI), hepatosomatic index (HSI), viscerosomatic index (VSI) and condition factor were calculated using standard methods and relationships [4]. Further, four fish were chosen at random from each tank, and after collecting blood, their liver and viscera were sampled and weighed. The dissected fish were returned to a bag (minus the blood) and kept frozen until further analysis. The remaining fish were returned to their respective tanks for a further one-week period of feeding with no EMS event prior to the application of AC stress below.

### 2.4. Chemical Analysis of Diets and Body

The proximate composition of diets and body samples was measured by AOAC methods [23]. Briefly, crude protein was determined using the Kjeldahl method and an automatic Kjeldahl system (Kjeltec Analyser unit 2300, FOSS, Hilleroed, Denmark). The Soxhlet extraction method was used to determine crude lipids (Soxtec 2050, FOSS, Effretikon, Switzerland). Moisture was determined gravimetrically by drying samples in a 105 °C oven for 12 h. The ash was determined gravimetrically using a muffle furnace (Model K, Nabertherm GmbH, Lilienthal, Germany) at 550 °C for 4 h.

Nitrogen-free extract plus fibre, representing carbohydrate, was calculated using the formula: carbohydrate = 100 − (protein + lipid + ash + moisture) [24]. Gross energy of the diet was calculated according to the following formula of the National Research Council [25]:

Energy (MJ/kg) = (protein × 23.6 kJ/g) + (lipid × 39.5 kJ/g) + (carbohydrate × 17.2 kJ/g)

#### 2.4.1. Collection of Blood and Sample Preparation

The serum from four fish from each tank was tested to measure haematology, immune response, blood biochemistry, antioxidants and serum enzymes at the end of the experiment (week 9 and week 10). Fish were sedated with clove oil (50 ppm) to decline stress in fish. Then, samples were quickly collected via caudal vein venipuncture with a sterile 5 mL syringe. Blood was refrigerated for 2 h to form the serum. Finally, the blood tube was centrifuged at 3000× *g* at 4 °C for 2 h to separate the serum and then stored at −20 °C until further analyses.

#### 2.4.2. Hematology Analysis

Red blood cells (RBCs) and white blood cells (WBCs) were counted in a Neubauer hemocytometer and the Neubauer chamber, respectively, as described before [2,26]. Further, the haemoglobin (Hb) and hematocrit (Ht) were determined by cyanmethemoglobin and the microhematocrit method [2,27]. Mean corpuscular volume (MCV), mean corpuscular haemoglobin (MCH), mean corpuscular haemoglobin concentration (MCHC) [28] and blood performance [29] were calculated according to the below formula:

Mean corpuscular volume (MCV) (fl) = (Ht/(RBC 106/mm^3^)) × 10

Mean corpuscular haemoglobin (MCH) (pg) = Hb/RBC 106/mm^3^ × 10

MCHC = Hb/Ht × 100

Blood Performance = Ln Hb (g/dL) + Ln Ht (%) + Ln RBC (∗10^5^/mm^3^) + Ln WBC (∗10^3^/mm^3^) + Ln total protein (g/L)

#### 2.4.3. Blood Biochemistry, Antioxidant Enzymes Activities, Serum Enzymes and Cortisol

Plasma biochemical parameters, glucose, total protein (TP), albumin, globulin, high-density lipoproteins (HDL), low-density lipoproteins (LDL), cholesterol, triglycerides, lactate, alkaline phosphatase (ALP), lactate dehydrogenase (LDH), aspartate transaminase (AST) and alanine aminotransferase (ALT) were analysed using commercial clinical investigation kits (Pars Azmoon Kit, Karaj, Iran). The antioxidant enzymes, including superoxide dismutase (SOD), catalase, glutathione peroxidase (GPx) and malondialdehyde (MDA) were measured using ELISA kits, according to the kit protocol (ZellBio, GmbH, Frankfurt am Main, Germany). Cortisol levels in serum were measured using commercial kits (iCHROMA, Chuncheon-si, Republic of Korea) and the protocol provided with the kit.

### 2.5. Non-Specific Immune Parameters

Gram-positive bacteria sensitive to the lysozyme enzyme method were used as a substrate to determine serum lysozyme (*Micrococcus lysodeikticus*) [30]. Alternative complement pathway haemolytic activity (ACH50) was determined by the haemolysis of rabbit RBCs (RaABC) [31]. Serum immunoglobulin, complement C3 (C3) and complement C4 (C4) levels were measured by the ELISA method using CUSABIO and MyBioSource kit companies (CUSABIO- CSB-E12045Fh- and CUSABIO, CSB-E09727s) based on the protocol available in the kit package. The complete methods for measuring these parameters were described earlier [32].

### 2.6. Acute Confinement Stress (AC Stress)

After ten weeks of the experiment, oscars exposed to AC stress based on our previous studies to test fish’s ability to tackle stressful situations [2,33]. After collecting samples at the end of week nine, ten fish per tank were biomass adjusted with three tanks per treatment. Then, the fish were fed as usual for one week and we applied AC stress at the end of week ten. The acute stress was a succession of netting all of the fish in each tank followed by a 30 s air exposure before being transferred to a plastic mesh bucket at a density of 120 g/L in their original tank for 5 h. Aeration was regularly carried out to prevent oxygen depletion and premature death. Following 5 h of stress, blood sampling and serum extraction were performed as previously described (three fish per tank [2]. The survival rate of fish after 48 h in various treatments is shown in Table 3.

### 2.7. Statistical Analysis

This experiment was based on a completely randomised design with nine treatments and three replications. Two-way ANOVA was used to investigate the “Diet Effect” and the “Stress-number Effect”. When the *p* value of interaction was not significant, treatments were compared in different stress numbers and three levels of FM replacement in pooled data (Table 4). When the interaction was significant, the original data were unpacked and treatments for each stress and FM level were compared (subsets in figures). Further, data were compared before and after AC stress to see which parameter was changed with AC stress in each treatment [2,20]. In all analyses, a significant difference between treatments was defined as a difference of 5% or less. SPSS (version 21.0 for Windows) was used to analyse the data.

## 3. Results

The main effects for this study were Diet Effect (different FM levels: 25, 18 and 11%) and “Stress-number Effect” (different stress that was 0-, 2- and 3-times stress). When the interaction was significant, we interpreted the original data as was explained in the statistical analysis section.

### 3.1. Growth Performance, Survival Rate and Body Composition

The effect of diet on growth parameters was not significant. However, the effect of stress was substantial, so that fish exposed to the 2Stress or Control groups had higher growth rates than the 3stress group (Table 3, 39.78 g and 38.92 g compared to 31.03 g) (*p* < 0.05). In the current study, FCR and feed intake were not changed by the FM level in diets or stress schedules. The survival rate after AC stress was affected by both diets and EMS numbers. Accordingly, the 2stress group had higher survival rates (70.0%) than the Control (52.2%) and the 3stress treatments (55.5%) (*p* < 0.05). Although fish fed dietary 11FM had the same growth rate, the survival rate after AC stress in this group (47.7%) was significantly lower than others (*p* < 0.05). None of the proximate composition parameters (protein, lipid, ash and moisture) were changed by stress schedules or dietary FM levels (Table 5).

### 3.2. Haematology and Blood Biochemistry

In the present study, both stress and diet effects were significant for haematological and blood biochemistry parameters. Accordingly, total protein and BP were lower in the 3Stress (3.44 and 14.17, respectively) group than the 0stress one (*p* < 0.05). The 2Stress had a higher value of total protein than the 3Stress group as well (*p* < 0.05).

**Figure 1:**
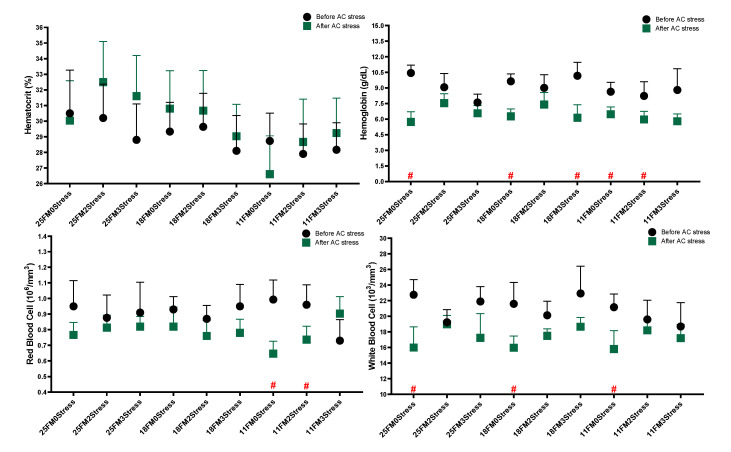

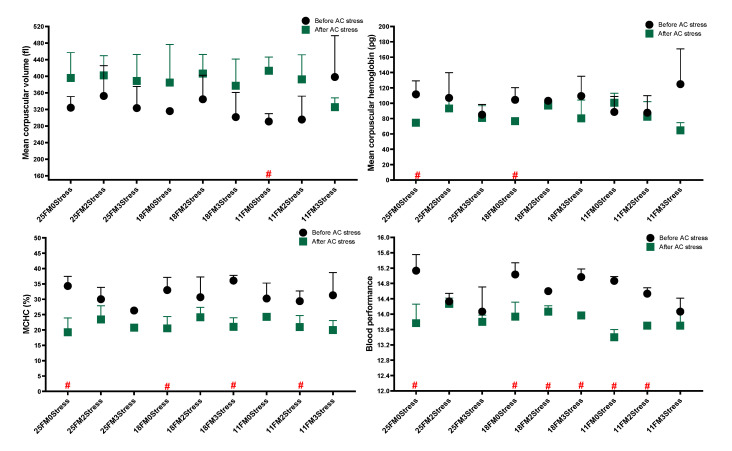
Haematological parameters of oscar fed experimental diets containing different levels of FM and exposed to secluded early mild stress for ten weeks plus data related to after acute confinement stress (AC stress). Values are represented by means and SDM of triplicate samples. Hash (#) with red colour indicates the significant difference in each treatment before and after AC stress according to the independent sample *t*-test (*p* < 0.05).

**Figure 2:**
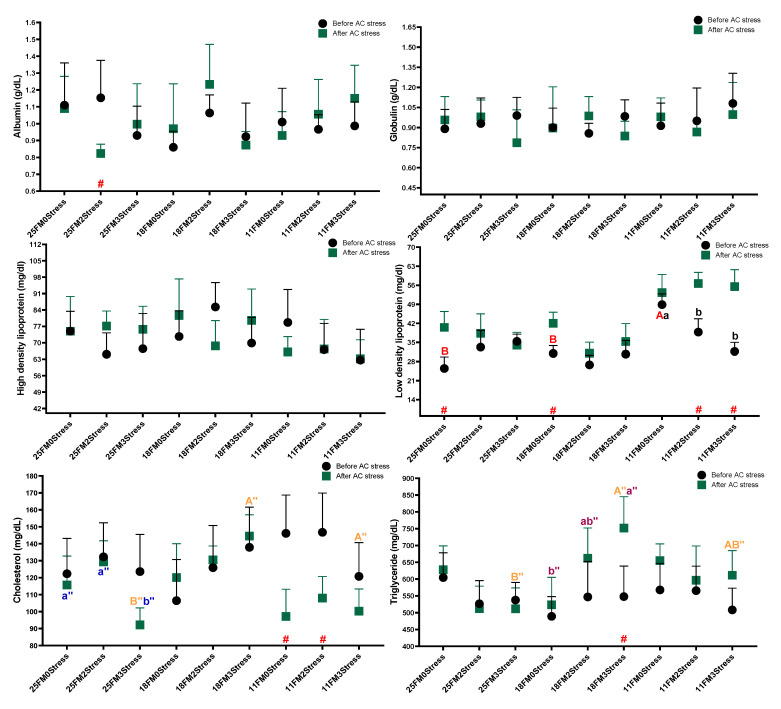
Blood biochemistry parameters of oscar fed experimental diets containing different levels of FM and exposed to secluded early mild stress for ten weeks plus data related to after acute confinement stress (AC stress). Values are represented by means and SDM of triplicate samples. Hash (#) with red colour indicates the significant difference in each treatment before and after AC stress according to the independent sample *t*-test (*p* < 0.05). The letters A, B and C with red, brown and orange indicate significant differences among 0Stress, 2stress and 3Stress groups after unpacking the data for the case that interaction was significant. The letters a, b and c in blue, pink and black indicate significant differences among the 25FM, 18FM and 11FM groups. The double quotation marks show that the comparison was among after AC stress groups.

In most haematological and blood biochemistry parameters after AC stress, FM levels in diets drove changes showing the key role of FM in stress responses (Figure 1). After AC stress, diet effects on Ht, WBC, BP, TP, glucose, cortisol and LDL were significant. In most of these parameters, the 11FM group had a lower value than others (*p* < 0.05). Cholesterol in the 11FM treatment was decreased after stress (*p* < 0.05) (Figure 2). After AC stress, the stress–number effect was only significant for cortisol, so the stressed groups had lower cortisol than the 0Stress group (*p* < 0.05). Figure 1 indicated that Hb (0Stress groups), RBC (11FM0Stress and 11FM2Stress), WBC (0Stress groups) and BP (0Stress groups, 18FM and 11FM groups) declined after AC stress (*p* < 0.05). Accordingly, the 3stress treatment also had a lower value of BP than others (*p* < 0.05).

### 3.3. Immune and Stress Responses

**Figure 3:**
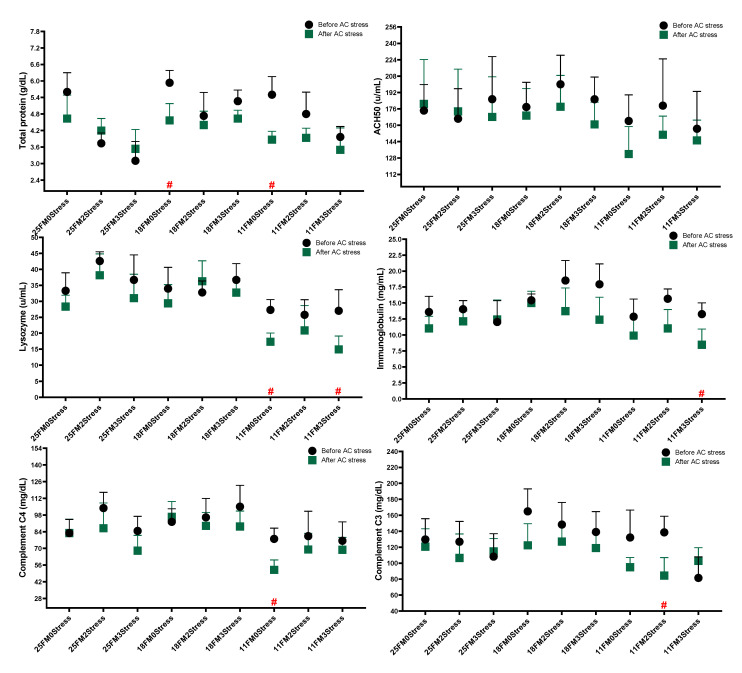
Immune response parameters of oscar fed experimental diets containing different levels of FM and exposed to secluded early mild stress for ten weeks plus data related to after acute confinement stress (AC stress). Values are represented by means and SDM of triplicate samples. Hash (#) with green colour indicates the significant difference in each treatment before and after AC stress according to the independent sample *t*-test (*p* < 0.05).

In this study, most of the immunological parameters were changed with the FM level in diets (Figure 3). Accordingly, the 11FM groups had lower values of lysozyme (26.70), immunoglobulin (13.93), C4 (78.23) and C3 (117.4) at the end of the study and after AC stress (*p* < 0.05). There was no significant difference in glucose, cortisol and lactate levels across nine groups at the end of the experiment. Cortisol levels in the stressed groups, especially the 2stress treatment, were lower than the Control. Further, three-time stressed fish had a lower value of C3 than other groups (*p* < 0.05). Lysozyme after AC stress in the 2stress group was higher than the Control (*p* < 0.05).

**Figure 4:**
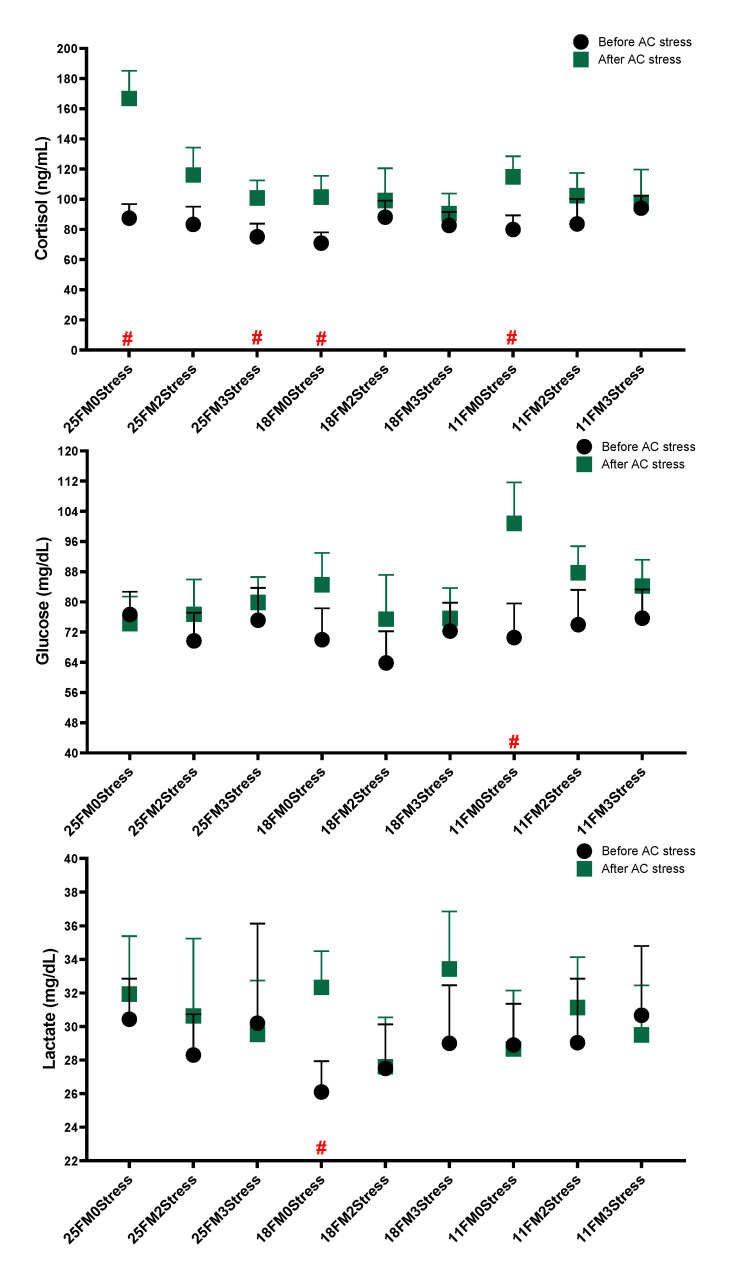
Stress response parameters of oscar fed experimental diets containing different levels of FM and exposed to secluded early mild stress for ten weeks plus data related to after acute confinement stress (AC stress). Values are represented by means and SDM of triplicate samples. Hash (#) with green colour indicates the significant difference in each treatment before and after AC stress according to the independent sample *t*-test (*p* < 0.05).

The data in Figure 4 indicated that in the 0Stress group, regardless of FM level, cortisol was increased, but the same output was not observed in stressed groups. Oscar fed 11FM had a higher glucose level after AC stress (*p* < 0.05). Cortisol levels in fish fed dietary 18FM had a lower value than the Control (*p* < 0.05).

### 3.4. Antioxidant Enzymes Activities

**Figure 5:**
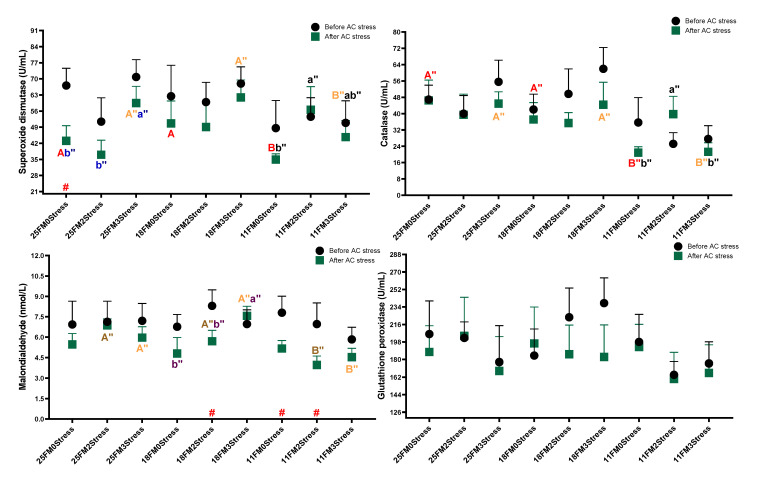
Antioxidant system parameters of oscar fed experimental diets containing different levels of FM and exposed to secluded early mild stress for ten weeks plus data related to after acute confinement stress (AC stress). Values are represented by means and SDM of triplicate samples. Hash (#) with green colour indicates the significant difference in each treatment before and after AC stress according to the independent sample *t*-test (*p* < 0.05). The letters A, B and C with red, brown and orange indicate significant differences among 0Stress, 2stress and 3Stress groups after unpacking the data for the case that interaction was significant. The letters a, b and c in blue, pink and black indicate significant differences among the 25FM, 18FM and 11FM groups. The double quotation marks show that the comparison was among after AC stress groups.

In the present research, while the stress effect was not significant for antioxidant enzymes, the FM effect was significant for SOD, CAT and GPx (*p* < 0.05) (Figure 5). Oscar fed the dietary 11FM had a lower value of these parameters (51.0, 29.49 and 179.5) as compared to other groups (*p* < 0.05). After AC stress, the interaction effect for SOD, catalase and MDA was significant and a variation with no specific trend in data was observed (*p* < 0.05). It shows that both diet and stress schedules affected these parameters in different directions.

### 3.5. Liver Enzymes

**Figure 6:**
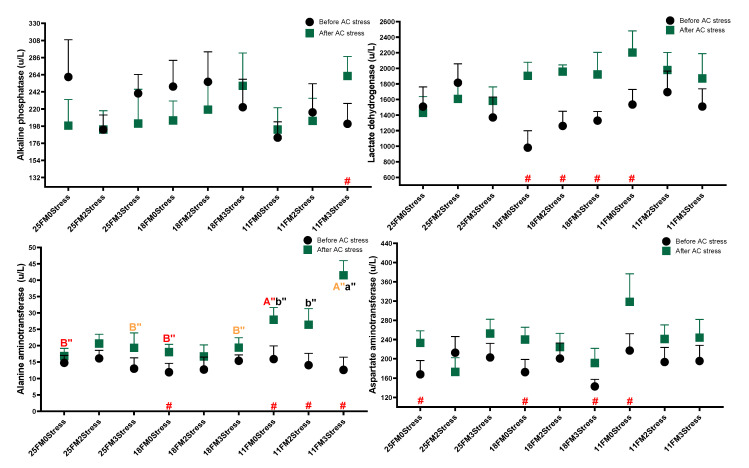
Liver enzyme parameters of oscar fed experimental diets containing different levels of FM and exposed to secluded early mild stress for ten weeks plus data related to after acute confinement stress (AC stress). Values are represented by means and SDM of triplicate samples. Hash (#) with green colour indicates the significant difference in each treatment before and after AC stress according to the independent sample *t*-test (*p* < 0.05). The letters A, B and C with red, brown and orange indicate significant differences among 0Stress, 2stress and 3Stress groups after unpacking the data for the case that interaction was significant. The letters a, b and c in blue, pink and black indicate significant differences among the 25FM, 18FM and 11FM groups. The double quotation marks show that the comparison was among after AC stress groups.

There were no specific trends or changes in liver enzymes with EMS numbers or FM replacement. After AC stress, the results were more meaningful and feeding oscar with the 11FM diet increased ALP, LDH and AST levels (*p* < 0.05). Further, AST in the 3stress treatment was higher than the other groups (Table 4) (*p* < 0.05). Figure 6 shows that AC stress increased ALT in the 11FM group (*p* < 0.05), which can be a sign of unhealthy status, which is matched with other physiological parameters mentioned in the last sections.

## 4. Discussion

### 4.1. Growth Performance and Survival Rate

Although stress has been studied for a long time as an adverse driver of growth and immunity, fewer studies tested the potential positive impact of EMS. The EMS prepares fish for future stressful situations, like a preparedness. This study is a follow-up study about EMS and its interaction with different FM levels. Esmaeili et al. (2022) indicated that EMS during the experiment (two out of ten weeks) improved stress responsiveness and survival after AC stress in oscars. Further, in that study, the interaction of EMS with diet lipid levels was tested, and results showed that oscar fed a high-lipid diet (180 g/kg) had a greater survival rate than those fed low-lipid diets. They reported both stress numbers and lipids in diets play a key role in survival rate after AC stress. In another investigation of this project, dietary protein levels and EMS were tested [20]. Two-times stress interestingly outperformed the other treatments in terms of growth and health indicators. They also tested 510 and 380 g/kg of protein in diets, and the first one resulted in a higher growth rate and immunity [20]. In these two studies [2,20], like the present research, oscars with approximately the same size, completely the same experimental conditions and design (in terms of EMS schedules) were used. The reason was to find which EMS schedule was better in terms of growth and health status. Based on the previous studies’ results, 450 g/kg and 180 g/kg of protein and lipid, respectively, were considered as optimum dietary requirements for the present research using oscar. This study was set up to understand how oscar responds to the FM replacement and whether the level of substitution (FM level) can interact with the EMS events, eventually affecting growth and health.

A higher survival rate in the 2stress group showed that more than two weeks of stress (out of ten) is not healthy for fish even though the growth rate was the same across treatments. Studies on EMS on this kind of schedule are limited to our previous studies. In those studies, the 2stress group was similarly the best treatment regarding the survival rate in oscars. Fish fed dietary 11FM had a lower survival rate after AC stress. Previous studies showed that lipid but not protein levels had a significant role in survival rate. Altogether, the crucial role of quantity and quality of dietary nutrients in fish health and stress response of fish was highlighted. This issue is less focused on research and farms, and it is wrongly believed that if fish grow well, they can also tolerate stress well. Therefore, it is highly recommended to test this hypothesis in different species and investigate growth and stress responsiveness interactions.

Marine-based ingredients like FM and fish oil are limited in their global supply, and therefore, providing them for the aquaculture industry is a major challenge. The FM production in 2018 was six million tonnes [34]. The price of FM is projected to increase up to 30 per cent by 2030 due to solid demand [34]. Until now, few studies have been conducted on FM replacement in ornamental fish species [35,36,37,38,39,40] and despite its potential relevance, no investigation in oscar. Decreasing FM level to a low level can potentially impair growth and fish health and therefore finding the optimum level is essential to improve the aquaculture sustainability of relative species. In the present study, the FM level had no significant effect on fish growth, meaning that the oscar eats and grows well with even 110 g/kg of FM and 60 g/kg of fish oil in their diets. Therefore, oscars readily accept diets with low FM levels when enough animal-based proteins are provided. However, there is no study on FM replacement with plant-based ingredients to see how oscar responds to declined FM levels in their diets. There are numerous studies that have shown that the FM level can affect the survival rate of fish after acute stress. For example, Chinese hooksnout carp (*Opsariichthys bidens*) fed MBM or BM (180 g/kg diet) substituting FM had higher mortality after 96 h of exposure to ammonia [41]. When red sea bream (*Pagrus major*) were fed a high-level soy protein concentrate diet and exposed to a freshwater stress test [42], and Ussuri catfish stressed to *Aeromonas hydrophila* with feeding cottonseed meal [43], lower survival rates were observed. Further, gibel carp (*Carassius auratus gibelio*) fed fermented moringa against *A. hydrophila* [44] showed the same trend.

In the present study, all the diets were palatable even at higher FM substitution levels and there was no negative effect on feed intake. The amino acids (Table 1) and fatty acids (unpublished data) profiles of the diets did not differ among treatments. The survival rate after nine weeks was at an acceptable level (91%) for all treatments. These reasons are evidence for not observing any significant difference in growth data, even based on lower FM levels. With matching growth rate and survival after the AC, it should be noted that fish health and welfare should be considered as well. The same results were observed in previous FM replacement by MBM in rainbow trout and ammonia stress [22]. More research is required to test whether FM can be replaced in oscar with alternative plants or other novel protein resources.

### 4.2. Body Composition

The proximate composition of tissues in fish can be changed by size, age, gender, quality of water, season, etc., but diet is the main driver [45]. Growth and body composition usually can have a direct relationship with each other, and it can be hypothesised that when the metabolism of protein and lipid is on the standard level, no change in growth would be observed. The lack of any changes in the present study is in line with recent works in terms of EMS [2,20] and inconsistent in terms of diet effect [20]. Interestingly, oscar is more sensitive to protein quantity than quality, so in the past study [20], protein content in the body was changed by diet. Zare et al. (2022) indicated that oscar fed 510 g/kg had a higher value of protein content compared to those fed 380 g/kg of protein in their diets. Similar to our results, some studies indicated no change in body composition when FM was replaced with MBM without any adverse effect on growth [13,14,46,47]. However, in these investigations, higher levels of MBM caused decreased growth and, accordingly, a decline in protein levels of the body. This is further evidence that growth and body composition under certain circumstances follow the same trend in FM studies.

### 4.3. Haematology and Blood Biochemistry

Hematology and blood biochemistry change when fish are exposed to stress and or changes in nutritional status. In most cases, these changes tend to decrease with different stresses [29]. In previous research, haematological parameters such as Ht, Hb, RBC, TP and BP were changed with protein levels in diet so that fish fed higher protein levels in diet (51%) had higher value compared to those fed 38% protein in their diet [20]. Further, Hb and BP changed with diet and EMS and oscars fed dietary with 18% lipid than 10% had higher Hb levels [2] and four-time EMS decreased BP as well [2]. Esmaeili et al. (2017b) indicated that haematological parameters and blood chemistry followed the trend of growth and that rainbow trout fed dietary 260 g/kg of MBM had the same profile of haematological (Hb, Ht, RBC, WBC, MCH, MCV, MCHC) and blood biochemistry parameters (TP, cholesterol, triglyceride) as the Control group (460 g/kg of FM). Similarly, no significant difference in HT, Hb, TP or glucose levels with using MBM to replace FM (526 g/kg of FM in the Control diet) up to 35% in the diets of spotted rose snapper (*Lutjanus guttatus*) was observed [48]. Conversely, a decrease in TP, albumin, cholesterol and triglyceride values in olive flounder (*Paralichthys olivaceus*) when 30% of FM (650 g/kg of FM in the Control diet) was replaced with MBM without decreasing growth was reported [49].

Like previous studies on this topic in oscar [2,20] the EMS groups had lower cortisol than the unstressed (0Stress) treatments. Cholesterol in the 11FM treatment was decreased after AC stress and showed lipid possibly was used for energy in this group. Although 110 g/kg of FM (11FM) was enough to provide sufficient growth in oscar, the haematological and blood biochemistry parameters were negatively affected. When fish are exposed to acute stress, the ability of fish to tackle this situation and keep their metabolism and these parameters stable can eventually end up with a greater survival rate. We clearly observed this phenomenon in this study and other research [50], and those with higher survival rates had fewer variations in these parameters.

High LDL is connected to an enhanced risk of cardiovascular diseases in mammals [51]. TP is a component of blood that is an indicator of protein levels and any change in the quantity or quality of proteins can change this parameter. Past studies have shown that high levels of FM replacement can decrease TP and increase LDL levels. For example, when FM was replaced with soybean meal in flounder [52], poultry by-product meal in hybrid grouper (*Epinephelus lanceolatus* ♂ × *Epinephelus fuscoguttatus* ♀) [53] and sacha inchi meal in tilapia (*Oreochromis niloticus*) [54], these results were observed. However, in contrast, these same results are not always concluded in other FM replacement studies [41,55].

The stress–number effect was not significant for haematological parameters after the AC stress, showing that EMS was mild and did not negatively affect fish haematology and health. These results are unlike our previous studies [2,20], where the four-times stress event was detrimental for fish but not two times. The BP parameter was introduced as a new haematological factor for aquaculture studies, consisting of the RBC, WBC, Ht, Hb and TP [42]. When the survival rate was matched with BP, this parameter was a reliable parameter and oscar with lower values of BP was weaker and less healthy. BP has been a reliable growth and health biomarker in previous works in oscar from this project [2,20]. Similarly, a lower BP was detected when fish were fed too much soybean [56], carbohydrate [57] and MBM [35].

### 4.4. Immune and Stress Response

Gut contains more than 70% of the immune system components in fish, and as a result, diet composition directly affects the immune response [58]. In the present research, immune parameters were more affected by FM levels than EMS schedules. These results may be connected to a lower survival after AC stress, and in the 11FM group, immunity was suppressed. In the only study related to MBM, lysozyme, immunoglobulin, C4 and C3 did not change with FM replacement in fish [41]. When the level of soy concentrate increased in the grouper diets, C3 and C4 contents showed an increasing trend at the start and then decreased [59]. As observed, there are many variations in the literature, and it is difficult to make any solid conclusions. The result may depend on fish species, FM level and alternative proteins employed. More investigations are required to see how these immune parameters can be changed with an animal by-product and how this eventually affects the growth rate.

AC stress suppressed the immunity of the 11FM0Stress group, which had no applied EMS schedules and was fed the 11FM diet. Other parameters also showed that these two factors (without EMS and feeding fish with 11FM diets) resulted in some adverse effects on oscar. Further, three-times stress did not change immunity of oscar. Unlike these data, our previous research showed the suppression of the immune system by four-times stress in oscar [2,20]. However, Zare et al. (2022) reported that immunoglobulin, C4 and C3 were affected by protein levels. By matching these two studies, it can be hypothesised that the immune system is more sensitive to protein quality and quantity. This result is further confirmed by the fact that most immune components have protein structures. The extent to which stress exposure is harmful and affects immunity, however, varies depending on the severity of the stress, the fish species, nutritional history and the type of stress [20].

The release of cortisol, glucose and lactate are the first and second responses to stress in fish, respectively. Information regarding the severity, time of stress and recovery period of the stress response can be provided by monitoring these parameters [60]. One of the main hypotheses for this study was that EMS and its interaction with FM levels in diets could prepare oscar to tolerate AC stress better. There was no significant difference in glucose, cortisol and lactate levels across nine groups at the end of the experiment. Interestingly, cortisol levels in the EMS group, especially the 2stress treatment, were lower than the Control, which is matched with previous investigations [2,20]. We deliberately selected fish with the same size, same experimental condition, stress schedule and AC stress among these three studies to see which EMS schedule has the best performance in terms of cortisol responsiveness and survival rate after AC stress. EMS alleviated AC stress, resulting in a higher survival rate in 2Stresses (70.0%); cortisol was not increased in this group. This demonstrates that the cortisol response of the fish was improved in EMS groups. The improved stress response can increase fish growth and survival, which is critical for aquaculture welfare. This important finding, confirmed in both of our studies, has now concluded that fish in the 2Stress group will have better stress responsiveness than those fish with no EMS exposure during the experimental period.

Increased cortisol response with an increasing level of MBM in the diets of rainbow trout was previously observed [22]. Esmaeili et al. (2017a) also reported that fish exposed to acute ammonia stress and fed a high level of MBM in diets had a lower survival rate and more elevated cortisol and glucose, which is in line with the present study. Other studies have shown that changes in stress markers can be induced with different used ingredients in diets. For example, in gilthead seabream (*Sparus aurata*) fed a diet with plant-based ingredients, higher cortisol and glucose levels were detected over the Control diet [61]. However, no change was observed in these markers in other FM replacement fish studies [62].

### 4.5. Antioxidant Enzymes Activities

Many markers have been used for monitoring fish under stressful or nutritional interventions. Antioxidant enzymes (SOD, CAT, GPx and MDA molecules) are among the most commonly applied markers [20]. These molecules defend cells against uncontrolled oxidative reactions that cause superoxide and H_2_O_2_ radical damage [63]. In the present research, oscar fed dietary 11FM had a lower value of antioxidant enzymes, which are matched with a lower survival rate in this treatment. These results are consistent with other published works when FM was replaced by mealworm (*Tenebrio molitor*) meal in hybrid grouper [64], MBM in Ussuri catfish [65], BM in black carp (*Mylopharyngodon piceus*) [66] and silkworm pupae meal in Jian carp (*Cyprinus carpio*) [67]. However, no change in the antioxidant activities of fish was observed with FM replacement with black soldier fly [68] and algal meals of *Schizochytrium limacinum* and *Nannochloropsis salina* [69].

In our previous studies, antioxidant enzyme activities were not changed with protein or lipid levels in diets [2,20]. However, in those studies, the 2Stress group of fish was the best treatment regarding antioxidant parameters, and these data were consistent with the current experiment. Many studies have found that increased antioxidant activity is associated with better health, growth and physiological condition in fish [63]. Lower survival in the treatment with lower antioxidant activity (11FM) following AC stress can be due to decreased resistance to oxidative stress and the maintenance of the antioxidant-reactive oxygen species balance. Further, there was no link between antioxidant activity and growth in this study, which is similar to earlier investigations [70,71,72]. The reason for higher antioxidant enzyme activities in fish fed diets with higher FM levels can be the fact that enough/optimum amino acids were provided to synthesise these enzymes and ensure homeostasis in the antioxidative system, resulting in improved growth and health. However, it should be noted that the amino acid profile of diets likely met the requirement of oscar fish for growth but perhaps not for optimum antioxidant enzymes.

### 4.6. Liver Enzymes

Liver enzymes or serological enzymes such as LDH, ALP, AST and ALT are frequently examined to monitor liver physiological status under different stressful or nutritional situations. Consistent with the growth data, there was no specific trend and change with EMS or diet in liver enzymes, illustrating that the liver was in a healthy condition. In both previous studies from our laboratory, the diet effect (lipid or protein) was insignificant for liver enzymes in oscar. However, survival after AC stress was lower in the 11FM group, and this goes against the observed alterations in previous studies. More studies are required in this area of research to comprehensively understand the effects of EMS and ingredients on liver enzymes in different fish species. Similarly, FM replacement did not change liver enzymes in fish, despite growth in those studies being affected [73] by feeding soybean meal [52,74], single-cell protein [75] and black soldier fly [76]. Conversely, elevated ALT and AST levels with FM replacement were observed [42,64,77,78,79]. These findings show that the responses of fish liver enzymes to FM levels in diets vary depending on fish species or other parameters such as size and diet composition. The overall results of the current liver enzyme analyses, combined with other physiological and biometrical parameters mentioned in the last sections, showed that the 11FM group might be unable to cope with AC stress effectively. Many past studies have reported increases in liver enzymes in response to various stresses [80,81,82,83,84]; however, it can be hypothesised that no or low change in the levels of liver enzymes is a positive sign of fish health and the ability to deal with stress. More research is required to demonstrate the effect of EMS on the liver’s physiological status.

## 5. Conclusions

The present research indicated that oscar could effectively utilise a diet formulated with 110 g/kg of FM, 200 g/kg of MBM and 110 g/kg of blood meal without any decreased growth (11FM group). However, this group had a lower survival rate after AC stress compared to other groups. Further, fish exposed to the 3Stress schedule had lower growth and survival rate than others, showing that three-week stress in a ten-week period is excessive for oscar. We observed that the scheduled stress of two weeks out of ten weeks does not harm but potentially improves fish health, survival rate and stress response on the parameters measured. After AC stress, the 3Stress group had the lowest value of C3 and the highest AST levels. The 11FM group had the lowest content of BP, TP, lysozyme, C4, C3, immunoglobulin, SOD, catalase, GPX and the highest values of LDH and AST. The lowest survival rates after AC stress can be connected to these changes. Considering all these parameters, replacement FM with MBM up to 28% (180 g/kg of FM, 130 g/kg of MBM) is recommended without any adverse effect. This study is further evidence that the 2Stress group was the best-scheduled EMS for oscar. To demonstrate the various mechanisms of EMS responses and nutritional history in fish, more programmed stresses and measuring more parameters at the classical and molecular levels are required.

## Figures and Tables

**Table 1:** Formulation and proximate analyses of the experimental diets replaced fish meal with meat and bone meal.

Ingredient	25FM	18FM	11FM	
g/kg, as-fed basis				
Corn meal	121.6	103.6	86.6	
Blood meal	80	95	110	
Fish meal	250	180	110	
Meat and bone meal	60	130	200	
Lysine	0	2	3	
Methionine	0	1	2	
Other ingredients ^†^	488.4	488.4	488.4	
Proximate composition (g/kg dry matter)				
Crude protein	445.1	450.0	448.9	
Crude lipid	182.4	175.1	171.3	
Ash	65.0	67.5	67.1	
Carbohydrate ^††^	199.2	196.4	194.5	
Moisture	108.3	113.0	111.2	
Gross energy (kJ/g) ^‡‡^	21.14	20.83	20.98	
Amino acids (g/kg, as-fed basis)				Requirements ^#^
Arginine	23.7	20.8	24.1	15
Histidine	8.4	7.6	8.2	8
Isoleucine	17.5	19.3	18.0	11
Leucine	30.3	28.7	29.7	15
Lysine	26.4	26.0	27.8	24
Methionine + Cysteine	11.2	11.3	11.0	10
Phenylalanine	20.9	22.1	21.2	9
Tyrosine	13.2	12.9	13.5	9
Threonine	18.1	18.5	19.4	11
Valine	21.8	22.2	22.7	12

^†^ Other ingredients: Soybean meal 240 g/kg; wheat gluten 85 g/kg; fish oil 60 g/kg; soybean oil 70 g/kg; dicalcium phosphate 3 g/kg; mineral premix 10 g/kg; vitamin premix 10 g/kg; antifungus Toxiban premix 10 g/kg; antioxidant, butylated hydroxytoluene (BHT) 0.2 g/kg; phytase 0.2 g/kg. Diet components were purchased from from the Animal & Aquatic Feed store (Arak, Markazi, Iran). 1 kg Mineral Supplementation contained: co, 100; I, 400; se, 20; Zn, 10,000; Fe, 6000; Cu, 600; Mn, 5000. 5 kg Vitamin Supplementation 0.5% contained: vitamin A 80,000 IU/kg; vitamin D3 2000 IU/kg; vitamin k 20 mg/kg; thiamin 60 mg/kg; riboflavin 60 mg/kg; pyridoxine 100 mg/kg; pantothenic acid 150 mg/kg; niacin 300 mg/kg; biotin 2 mg/kg; folic acid 20 mg/kg; vitamin B12 0.1 mg/kg; inositol 300 mg/kg; ascorbic acid 600 mg/kg; choline chloride 3000 mg/kg. ^††^ Carbohydrate = 100 − (crude protein + crude lipid + ash + moisture). ^‡‡^ Estimated gross energy was calculated based on 1 g crude protein being 23.6 kj, 1 g crude lipid being 39.5 kj and 1 g carbohydrate being 17.2 kj. NRC (2011). ^#^ Based on rainbow trout requirements. NRC (2011).

**Table 2:** The treatments of the present study under fish meal replacement, scheduled early mild stress and AC stress at the end of experiment.

	25FM, without Stress	25FM, 2-Time Stress	25FM, 3-Time Stress	18FM, without Stress	18FM, 2-Time Stress	18FM, 3-Time Stress	11FM, without Stress	11FM, 2-Time Stress	11FM, 3-Time Stress
Week 1	WS	WS	WS	WS	WS	WS	WS	WS	WS
Week 2	WS	Stress	Stress	WS	Stress	Stress	WS	Stress	Stress
Week 3	WS	WS	WS	WS	WS	WS	WS	WS	WS
Week 4	WS	WS	WS	WS	WS	WS	WS	WS	WS
Week 5	WS	WS	WS	WS	WS	WS	WS	WS	WS
Week 6	WS	WS	Stress	WS	WS	Stress	WS	WS	Stress
Week 7	WS	WS	WS	WS	WS	WS	WS	WS	WS
Week 8	WS	Stress	Stress	WS	Stress	Stress	WS	Stress	Stress
Week 9	WS	WS	WS	WS	WS	WS	WS	WS	WS
Calculation of weight of all fishes, growth, sampling of blood and other factors									
Week 10	WS	WS	WS	WS	WS	WS	WS	WS	WS

WS: without stress; final stress (AC stress) and sampling for serum and haematological parameters.

**Table 3:** Growth performance of oscar (*Astronotus ocellatus*) fed experimental diets under fish meal replacement with meat and bone meal and subjected to varying numbers of stresses.

	25FM0Stress	25FM2Stress	25FM3Stress	18FM0Stress	18FM2Stress	18FM3Stress	11FM0Stress	11FM2Stress	11FM3Stress
Initial weight (g)	5.20 ± 1.25	5.33 ± 1.11	5.77 ± 1.12	4.47 ± 0.71	5.40 ± 1.35	4.72 ± 0.44	5.25 ± 1.29	5.07 ± 0.40	5.57 ± 0.65
Final weight (g)	47.17 ± 7.47	40.57 ± 6.84	36.23 ± 6.57	43.08 ± 4.40	50.64 ± 9.35	35.47 ± 7.40	44.03 ± 5.78	41.36 ± 6.95	37.46 ± 6.06
SGR (%/day)	3.52 ± 0.20	3.23 ± 0.20	2.92 ± 0.25	3.61 ± 0.38	3.57 ± 0.18	3.18 ± 0.35	3.40 ± 0.56	3.32 ± 0.09	3.02 ± 0.18
FCR	2.06 ± 0.18	2.24 ± 0.21	2.28 ± 0.31	2.20 ± 0.37	2.20 ± 0.29	2.44 ± 0.44	2.18 ± 0.25	2.20 ± 0.27	2.34 ± 0.35
DFI (%/day)	5.27 ± 0.52	5.46 ± 0.92	5.25 ± 0.78	5.66 ± 1.02	5.66 ± 0.76	5.88 ± 1.26	5.41 ± 0.62	5.43 ± 0.65	5.51 ± 0.99
HSI (%)	2.30 ± 0.37	2.59 ± 0.52	2.52 ± 0.36	2.35 ± 0.54	2.32 ± 0.27	2.48 ± 0.16	2.98 ± 0.43	2.89 ± 0.34	3.04 ± 0.81
VSI (%)	6.17 ± 0.40	6.11 ± 0.51	6.20 ± 0.56	5.97 ± 0.32	6.20 ± 1.05	6.14 ± 0.66	6.30 ± 0.46	6.03 ± 0.57	6.20 ± 0.56
Condition factor	2.39 ± 0.17	2.06 ± 0.19	1.92 ± 0.12	2.21 ± 0.19	2.51 ± 0.13	1.89 ± 0.34	2.29 ± 0.57	2.09 ± 0.23	2.01 ± 0.32
Survival rate (%) ^#^	94.44 ± 6.36	94.44 ± 4.81	91.67 ± 4.17	93.06 ± 2.41	95.83 ± 7.22	91.67 ± 8.33	93.06 ± 6.36	91.67 ± 0.00	91.67 ± 4.17
Survival rate after AC (%)	56.67 ± 5.77	83.33 ± 11.55	63.33 ± 20.82	56.67 ± 5.77	73.33 ± 20.82	56.67 ± 20.82	43.33 ± 11.55	53.33 ± 15.28	46.67 ± 5.77

SGR: Specific Growth Rate = ((Ln W2 − Ln W1)/63 days)) × 100. FCR: Feed Conversion Ratio = Dry feed consumed (g)/WG (g). DFI: Daily feed intake (%body weight.day^−1^) = 100 × feed consumed (g)/((initial weigh + final weight) × 0.5 × days). HSI: Hepatosomatic Index = (Liver weight (g)/Body weight (g)) × 100. VSI: Viscerosomatic Index = (Visceral weight (g)/Body weight (g)) × 100. CF: Condition Factor = (W2 (g)/Length^3^) × 100. ^#^ Survival rate (%) = (Number of fish in each group remaining at the end of experiment/initial number of fish: 24) × 100.

**Table 4:** The results of two-way ANOVA analysis with SPSS for measured factors. When the interaction was not significant, we compared the “Diet Effect” via Independent Samples *t*-Test and “Stress-number Effect” via Duncan’s new multiple range test for the case these effects were significant (*p* < 0.05). The non-significant parameters were not reported. The letters a, b and c indicate significant differences among treatments exposed to different stress numbers.

	*p*-Value				Main Effects (Mean)				
	Diet Effect	Stress–Number Effect	Interactions	25FM Diets	18FM Diets	11FM Diets	0stress	2stress	3stress
Final weight	0.790	0.033	0.409				44.75 a	44.19 a	36.39 b
Weight gain	0.666	0.019	0.429				39.78 a	38.92 a	31.03 b
SGR	0.260	0.031	0.974				3.51 a	3.37 a	3.03 b
Survival rate after AC	0.025	0.042	0.869	67.7 a	62.2 a	47.7 b	52.2 b	70.0 a	55.5 b
Total protein	0.109	0.001	0.347				5.68 a	4.42 b	3.44 c
BP	0.253	0.001	0.633				15.01 a	14.55 ab	14.17 b
Ht-stress	0.041	0.469	0.608	31.38 a	30.07 b	28.16 b			
WBC-stress	0.920	0.049	0.762	15.92 b	18.22 a	17.70 a			
BP-stress	0.025	0.127	0.658	13.93 a	13.99 a	13.62 b			
TP-stress	0.036	0.247	0.457	4.53 a	4.12 ab	3.77 b			
Glucose-stress	0.006	0.202	0.288	76.94 b	78.53 b	90.91 a			
Cortisol-stress	0.032	0.028	0.609	119.1 a	97.2 b	104.8 ab	118.8 a	105.8 b	96.2 b
LDL-stress	0.001	0.336	0.232	37.59 b	36.12 b	55.17 a			
Lysozyme	0.001	0.646	0.360	37.53 a	34.48 a	26.70 b			
Immunoglobulin	0.005	0.179	0.661	13.22 b	17.30 a	13.93 b			
Complement C4	0.034	0.440	0.510	90.44 ab	97.78 a	78.23 b			
Complement C3	0.036	0.041	0.608	121.6 b	150.8 a	117.4 b	142.3 a	137.9 a	109.6 b
Lysozyme-stress	0.001	0.048	0.837	32.50 a	32.80 a	17.72 b	25.01 b	31.79 a	26.22 ab
Immunoglobulin-stress	0.025	0.638	0.705	11.87 ab	13.71 a	9.80 b			
C4-stress	0.001	0.571	0.230	79.29 a	91.20 a	63.33 b			
C3-stress	0.032	0.770	0.835	113.9 ab	122.8 a	94.1 b			
SOD	0.020	0.218	0.321	63.1 a	63.4 a	51.0 b			
Catalase	0.001	0.090	0.145	47.48 a	51.22 a	29.49 b			
GPX	0.039	0.997	0.066	195.2 ab	215.1 a	179.5 b			
ALP	0.035	0.764	0.094	231.5 ab	242.0 a	199.9 b			
LDH	0.002	0.074	0.286	1564 a	1190 b	1579 a			
ALP-stress	0.198	0.047	0.496	199.2 b	205.8 ab	237.8 a			
LDH-stress	0.001	0.843	0.423	1540 b	1927 a	2017 a			
AST-stress	0.009	0.016	0.059	219.3 b	218.4 b	267.8 a	212.7 b	229.1 b	263.7 a

The interaction effect for LDL, cholesterol-stress, triglyceride-stress, SOD-stress, catalase-stress, MDA-stress and ALT-stress were significant which was unpacked and shown in figures.

**Table 5:** Carcass chemical composition (g/kg) of oscar (*Astronotus ocellatus*) fed experimental diets fish meal replacement with meat and bone meal and subjected to varying numbers of stresses.

	25FM0Stress	25FM2Stress	25FM3Stress	18FM0Stress	18FM2Stress	18FM3Stress	11FM0Stress	11FM2Stress	11FM3Stress
Protein	180.2 ± 18.5	186.9 ± 9.4	184.8 ± 6.5	180.0 ± 15.7	178.8 ± 11.8	181.9 ± 9.2	183.6 ± 14.4	175.1 ± 6.7	182.1 ± 2.3
Lipid	60.2 ± 5.8	63.1 ± 6.2	59.7 ± 7.2	60.2 ± 7.3	61.7 ± 6.4	61.1 ± 5.2	58.1 ± 4.5	61.6 ± 5.6	62.1 ± 3.9
Ash	30.6 ± 3.5	31.0 ± 5.5	30.0 ± 2.6	27.6 ± 3.8	31.6 ± 4.0	29.7 ± 2.1	30.7 ± 3.5	30.5 ± 2.5	28.7 ± 2.6
Moisture	686.7 ± 51.4	689.4 ± 38.5	672.2 ± 34.0	690.8 ± 55.4	678.6 ± 35.6	672.8 ± 47.8	683.9 ± 38.1	692.9 ± 44.4	697.8 ± 50.6

Values are represented by means ± SDM of triplicate tanks.

## Data Availability

Data available on request due to privacy/ethical restrictions (the data that support the findings of this study are available on request from the corresponding author. The data are not publicly available due to privacy or ethical restrictions).
